# Impact of the Oxidant Type on the Efficiency of the Oxidation and Removal of Iron Compounds from Groundwater Containing Humic Substances

**DOI:** 10.3390/molecules25153380

**Published:** 2020-07-25

**Authors:** Izabela Krupińska

**Affiliations:** Faculty of Civil Engineering, Architecture and Environmental Engineering, Institute of Environmental Engineering, University of Zielona Góra, 15 Prof. Z. Szafrana St, 65-516 Zielona Góra, Poland; i.krupinska@iis.uz.zgora.pl; Tel.: +48-68-3282560

**Keywords:** groundwater, organic substances, iron-organic complexes, aeration, chemical oxidation

## Abstract

Due to the coexistence of organic matter and iron in groundwater, a certain part of the iron is present as iron-organic complexes in the form of colloids and/or dissolved complexes. The study was conducted to evaluate the impact of the type of oxidizing agent: O_2_, Cl_2_, H_2_O_2_, or KMnO_4_, on the efficiency of the oxidation and removal of iron compounds from three groundwaters with significantly different contents and types of organic substances among which humic and fulvic acids occurred. This study shows that after the aeration and the oxidation with Cl_2_ and H_2_O_2_, the increasing content of dissolved hydrophilic organic substances containing aromatic rings in the raw water reduced the effectiveness of Fe(II) oxidation and the effectiveness of iron removal during the sedimentation process. This regularity was not found only when KMnO_4_ was used as the oxidant. After oxidation with H_2_O_2_, the highest number of organo-iron complexes and an increased concentration of dissolved organic carbon were found. High concentrations of organo-ferrous connections were also found in aerated water samples. The highest KMnO_4_ efficiency of removing iron and organic substances and reducing the color intensity and turbidity was due to the catalytic and adsorptive properties of the precipitated MnO_2_, which also improved the sedimentation properties of the resultant oxidation products.

## 1. Introduction

Groundwater components, in addition to iron and manganese affecting the method of their purification, include organic substances [1,2,3]. Organic substances naturally found in groundwater are a mixture of various organic compounds, including primarily humic substances [4]. Humic substances are macromolecular compounds with a molecular weight in the range from 500 to 100,000 Da. They are most often classified due to their solubility: humic acids, soluble in aqueous solutions of alkali, oxalate, and sodium fluoride; fulvic acids, soluble in water, alkali, alcohol, and mineral acids; hymatomelanic (ulmin) acids, soluble in ethanol. Humic acids are macromolecular compounds with a particle mass in the range of 50,000–100,000 Da and a diameter of 60–100 Ǻ, while fulvic acids are in the range from 500 to 2.000 Da and have a diameter of 20–30 Ǻ [5]. Fulvic acids compared to humic acids and humins reveal a structure richer in oxygen compounds, a lower sensitivity to pH, better solubility, lower molecular weight, and lower aromaticity. Fulvic acids, due to the large number of carboxylic, phenolic, and hydroxyl functional groups, show chelating properties, and they can play a key role in the mobility of metals [6]. According to Thurman, in groundwater, eighty-seven percent of dissolved organic substances (DOC) are fulvic acids, and only 13% are humic acids [7,8]. To characterize organic substances in natural waters, many researchers mark the absorbance specific SUVA_254_, i.e., the ultraviolet absorbance at 254 nm related to the content of DOC [9]. SUVA_254_ ≤ 2 m^2^/gC values indicate the presence of low molecular weight hydrophilic substances. SUVA_254_ values in the range of 2–4 m^2^/gC mean that hydrophilic and hydrophobic, as well as low and high molecular organic substances are found in water, while SUVA_254_ ≥ 4 m^2^/gC values indicate the presence of mainly an aromatic high molecular weight hydrophobic fraction [10,11,12,13,14,15,16]. Iron removal from groundwater is based on the oxidation of Fe(II) ions to Fe(III) and removal of the precipitated Fe(OH)_3_ by sedimentation and filtration. Iron(II) is oxidized with oxygen (Reaction 1) or other oxidants (potassium permanganate, ozone, chlorine).

2Fe^2+^ + 1/2O_2_ + 5H_2_O ↔ 2Fe(OH)_3_ + 4H^+^ (1)

In oxygen saturated water deprived of organic substances, the quick oxidation of Fe^2+^ to Fe^3+^ takes place. The necessary time for Fe^2+^ ions’ oxidation extends mainly along the concentration of Fe^2+^ and H^+^ in water and, to a smaller degree, the increase in water temperature. 

The kinetics of the oxidation are second order with respect to pH:

(2) $$\frac{-d\left[\mathrm{Fe}\left(\mathrm{II}\right)\right]}{\mathrm{dt}}=k[\mathrm{Fe}(\mathrm{II})][{\mathrm{OH}}^{-}{]}^{2}P_{O2}$$

where: k- reaction rate constant; [Fe(II)]—concentration of Fe(II) in water; [OH^-^]—concentration of hydroxide ions in water; P_O2_—oxygen partial pressure; t—oxidation time; dt—time interval. Generally speaking, a pH increase of one results in about a 100-fold increase in the rate of iron oxidation, so a higher pH results in more rapid oxidation [17]. Gonczarow et al. [18] proved that the half times of the oxidation reaction of Fe^2+^ to Fe^3+^, at an oxygen partial pressure equal to 21.3 kPa, extended greatly along the increasing concentration of hydrogen ions in treated water and for pH = 7, pH = 6, and pH = 5 amounted to approximately 4 min, 6 h, and one month. In natural waters where organic substances are present, at the same pH values, p0_2_, and concentration of Fe^2+^, the speed of iron oxidation is several times slower, which, according to some researchers [19], is caused by the stabilization of Fe^2+^ by organic compounds. 

**Figure 1:**
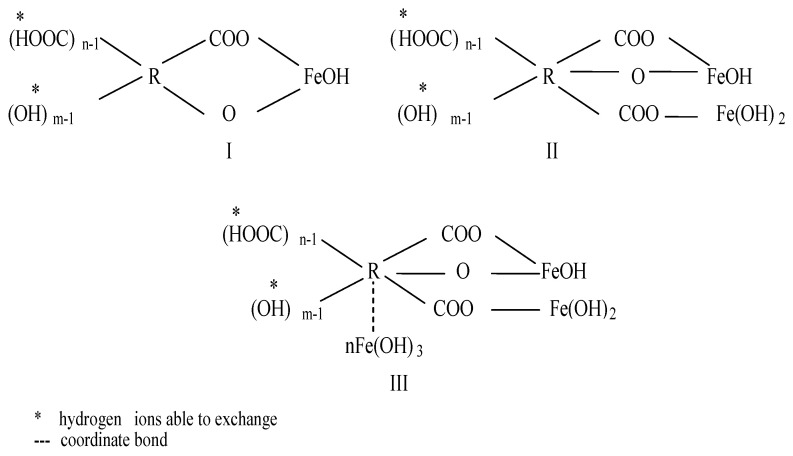
Iron-organic complexes [16].

Several researchers [16,17,18,19,20,21,22,23] suggested that the formation of chelate complexes with the humic substances and iron occurs as a result of an exchange reaction between the proton of carboxyl and phenol groups of the humic substances and iron hydroxo-complexes: [Fe(OH)]^2+^, [Fe(OH)_2_]^+^ (Figure 1). According Khatri et al. [24], the organically complexed iron is resistant to oxidation and is not readily removed by filtration.

During aeration of groundwater containing iron and organic substances, mainly humic substances, no easily sedimenting iron(III) hydroxide agglomerates are precipitated, but colloidal and colored iron-organic compounds dissolved in water are formed [1].The higher the pH, the more the oxidation process of the iron(II)-organic complex is retarded. For example, at pH 8, a decrease in the rate constant by a factor of 10 results in doubling the half life of the Fe(II)-organic complex with respect to oxidation [25,26]. According to Bratby [27], at pH ≥ 8.0, humic substances are completely dissociated. At pH = 4.6–4.9, the dissociation of proton starts from carboxyl groups, whereas at pH ≥ 8.0, protons dissociate from hydroxyl groups. According to numerous researchers [1,2,12,21], one of the reasons for iron stabilization by organic substances may also be the formation of the so-called protective colloids. Protective colloids have a hydrophilic character as a result of the adsorption of organic substances on Fe(OH)_3_. To remove iron-organic connections, it is not enough to use processes traditionally used for groundwater purification [22,23]. Therefore, various attempts are being made to intensify groundwater purification with increased organic content. One of the methods is the use of chemical oxidants [28]. The inclusion of the oxidation process in the technological system of water purification is justified only when the chemical oxidants used do not cause harmful inorganic and organic oxidation by-products, and the products of incomplete oxidation of organic substances will be removed from the water [29,30,31,32]. The standard potentials are a useful reference regarding the strength of an oxidant. However, the potential does not indicate how the oxidants will perform under field conditions. The oxidation potential is often used to determine relative effectiveness for oxidizing organic constituents. The hydroxyl radical has a standard oxidation potential of 2.8 V, while permanganate, chlorine, ozone, hydrogen peroxide, and oxygen have standard oxidation potentials of 1.68, 1.36, 2.1, 1.77 V, and 1.20 V, respectively [33]. Hydrogen peroxide is a more powerful oxidant than chlorine and potassium permanganate; however, in water, it is very slow to act, often too slow to be of practical application. Hydrogen peroxide is also a relatively weak oxidant with respect to organic compounds. This is due to the relatively low reactivity of H_2_O_2_ towards organic functional groups and the fact that in the presence of electrophilic compounds, it can react as a nucleophile (undergo substitution reactions), without manifesting oxidizing properties [34]. During purification of groundwater containing humic substances and iron compounds, chlorine compounds should not be used as oxidants due to the formation of colloidal iron compounds that are not retained on filter beds and due to the danger of chlorinated organic compounds [35]. The advisability of using other oxidants such as ozone and hydrogen peroxide is also disputable. The oxidation with ozone and hydrogen peroxide involves the transformation of the structure of humic substances by breaking large molecules into smaller ones, which destroys conjugated bonds determining the color, but also causes the formation of by-products of oxidation. These oxidation by-products include carboxylic acids, aldehydes, and ketones [36,37,38]. The oxidation efficiency is therefore only apparent because the decrease in color is not always accompanied by the release of iron ions from the complexes and their oxidation, as well as a decrease in the concentration of DOC in purified water. An alternative to ozonation may be the use of Fenton’s reagent. Fenton oxidation is an efficacious advanced oxidation processes and implies a catalytic degradation of hydrogen peroxide (H_2_O_2_) by ferrous iron (Fe^2+^) to form hydroxyl radicals (^•^OH) with high oxidative power. The produced ^•^OH after that oxidizes the organic substances [39]. According to literature reports [40], pH is a determinant factor in the Fenton process. At pH values in the range of 2.0–4.0, the highest concentration of Fe^2+^ occurs, and hydrogen peroxide is most stable at pH levels in the range of 3.0–4.0. For oxidation of Fe^2+^ stabilized by organic substances, according to the author, potassium permanganate should be preferred, because the oxidation process is supported additionally due to the sorption and catalytic properties of the precipitated MnO_2_ according to Reaction 3 [27,41,42,43,44].

3Fe^2+^ + MnO_4_^−^ + 7H_2_O ↔ 3Fe(OH)_3_ + MnO_2_ + 5H^+^ (3)

The colloidal MnO_2_ that appears has a positive charge with pH ≥ 8 and a vast specific surface area with good sorptive characteristics [25,38,43,44]. Compared with other chemical oxidants, potassium permanganate can markedly avoid the production of hazardous by-products [45]. The application of potassium permanganate in drinking water treatment is receiving great attention currently [46,47,48,49,50]. Another method suggested for treating groundwater contaminated with organic substances and iron compounds is the coagulation process with the use of aluminum coagulants [2,16,25,38,51,52]. Due to the coexistence of organic matter and iron in groundwater, a certain part of the iron is present as iron-organic complexes in the form of colloids and/or dissolved complexes, and the water is characterized by increased color intensity and turbidity. Effective treatment of such water has technological difficulties and is practically impossible in the case of traditional groundwater treatment processes. Therefore, researchers and responsible parties are constantly seeking new methods and optimizing the existing ones. This paper compares the efficiency of different oxidants such as oxygen, chlorine, hydrogen peroxide, and potassium permanganate in the oxidation and removal of iron compounds from groundwater significantly differing in the type and content of Natural Organic Matter (NOM) among which humic substances occur. This paper also determines the impact of the type of organic matter fraction, highly hydrophobic, slightly hydrophobic, and hydrophilic on the efficiency of groundwater purification in oxidation and sedimentation processes, with particular emphasis on the removal of iron compounds and the formation of iron-organic complexes.

## 2. Results and Discussion

### 2.1. Groundwater

**Figure 2:**
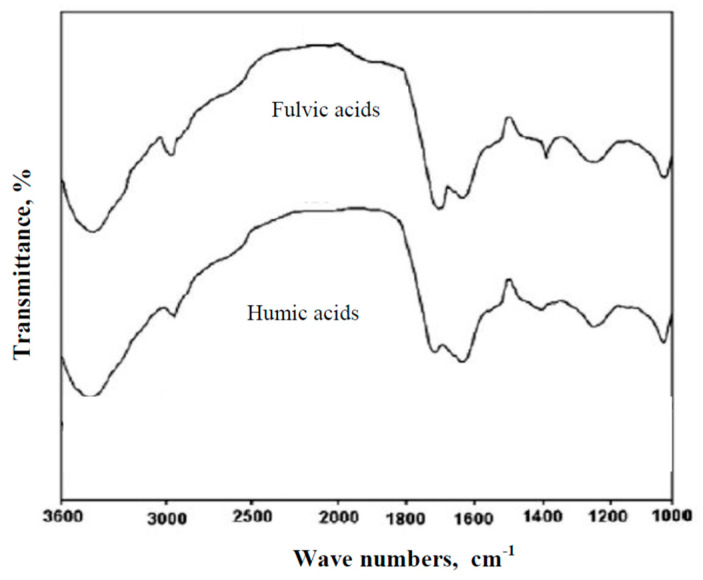
The IR spectra of humic and fulvic acids extracted from the groundwater.

Analysis of the IR spectra of organic substances extracted from raw waters showed that all raw water samples contained humic and fulvic acids. Figure 2 shows the IR spectra obtained for humic and fulvic acids extracted from the groundwater tested.

IR spectra shown in Figure 2 have a diversity of bands typical of humic substances such as humic and fulvic acids [5,53,54]. Absorption bands are in the regions ~3400 cm^−1^ (O-H stretch vibration due to alcoholic, phenolic, and acid groups), 2940–2900 cm^−1^ (aliphatic C-H stretching), ~1720 cm^−1^ (carboxylic acids, esters, ketone, and aldehyde structures give rise to the C=O stretching vibration), 1620 cm^−1^ (aromatic C=C, COO^−^, H-bonded C=O), ~1280cm^−1^ dominated by the C-O stretching vibrations and O-H deformations in carboxylic acids and acrylethers, and 1040 cm^−1^ (C-O stretching of polysaccharide). According to Frimmel [5], the spectra of fulvic acid is characterized by stronger absorption near 1720 cm^−1^, which implies the high carboxylate capacity. The spectrum of fulvic acid is also characterized by the absorption at ~1400cm^−1^ due to the O-H bonding vibrations of alcohols and carboxylic acids. Based on the IR spectra, it can be concluded that fulvic acid is more aliphatic while humic acid is more aromatic, which was also confirmed by other authors [5,55,56,57]. In raw water, the specific absorbance value in UV-SUVA_254_ was calculated on the basis of the measured UV absorbance at 254 nm and dissolved organic carbon content (DOC): 4.206, 3.565, and 2.783 m^2^/gC for Waters sample No. 1, 2, and 3, respectively. Based on the calculated SUVA_254_ values, it can be stated that in Water 1, there were mainly hydrophobic, aromatic, and high molecular DOC fractions, while in Waters 2 and 3, there was a mixture of hydrophilic and hydrophobic humic substances and other organic compounds of both low and high molecular weights [10,11,12,13,14,15]. Fractionation of organic substances present in the tested groundwater by means of filtration through a 0.45 µm filter and adsorption on Amberlite XAD7HP and XAD4 resins allowed determining the percentage of isolated fractions of organic substances. The characteristics of individual fractions of organic substances in the tested groundwater are presented in Table 1.

The largest (87%) share of the hydrophilic fraction was found in water sample No. 3 and the largest (60%) share of the hydrophobic fraction in water sample No. 1. The analysis of the obtained test results (Table 2) also showed that with the increase in the co-occurrence coefficient of organic substances and total iron in raw water (D and D’) and with the increase in the absorbance value in UV_254_, which indicates that among the dissolved organic substances, there were organic compounds containing aromatic rings, increased the amount of iron bound to organic substances, as well as the actual color of the water.

The analysis of the test results also showed that the amount of formed organic iron compounds and the actual color increased with the decrease of SUVA_254_ value in raw water, and thus with the increase in the water content of hydrophilic organic substances. On the other hand, the turbidity of water increased with the increase of Fe(III) content in raw water and with the decrease in the raw water absorbance value UV_254_, as well as the decrease of the co-occurrence coefficients of organic substances and general iron D and D’. The percentage of Fe(II) in total iron grew with the increase in absorbance value UV_254_ and with the increase in the values of the co-occurrence coefficients of organic substances and total iron D and D’. The regularities found indicate inhibition of the oxidation of Fe(II) by organic substances present in water, and especially by organic substances containing aromatic rings (Table 2). 

### 2.2. Oxidation Efficiency of Fe(II) by Oxygen and Chemical Oxidants

**Figure 3:**
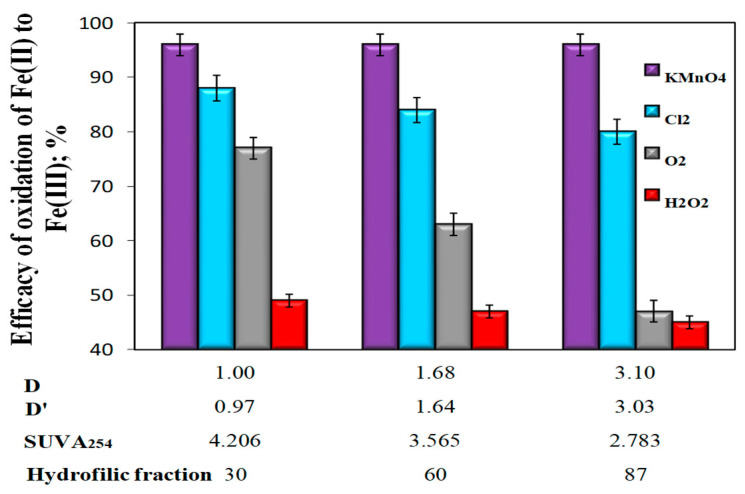
The impact of the type of oxidant and values of the co-occurrence coefficient of organic substances and total iron (D and D’), SUVA_254_ coefficient, and hydrophilic organic content (%) on the oxidation efficiency of Fe(II) to Fe(III).

The analysis of the obtained test results showed that the type of oxidizer used determined the oxidation efficiency of Fe(II) to Fe(III) and, in the case of aeration and oxidation with hydrogen peroxide and chlorine oxidant, the co-occurrence coefficients of organic substances and total iron in raw water D (TOC)/Fe_tot_) and D’(DOC/Fe_tot_) and the value of specific SUVA_254_. With the increase of the co-occurrence coefficients of organic substances and total iron in raw water (D and D’) and with the decrease of SUVA_254_, as well as with the increase of the content of hydrophilic organic substances, the oxidation efficiency of Fe(II) decreased, which was particularly evident in the case of aerated water samples (Figure 3). In water samples in which KMnO_4_ was used for the oxidation of Fe(II), there was no effect of the values of coexistence of organic substances and total iron in raw water D and D ‘oxidation efficiency of Fe(II) to Fe(III). There was also no effect of the specific absorbance value SUVA_254_, as well as the content of the hydrophilic organic fraction on the oxidation efficiency of Fe(II) (Figure 3). 

**Figure 4:**
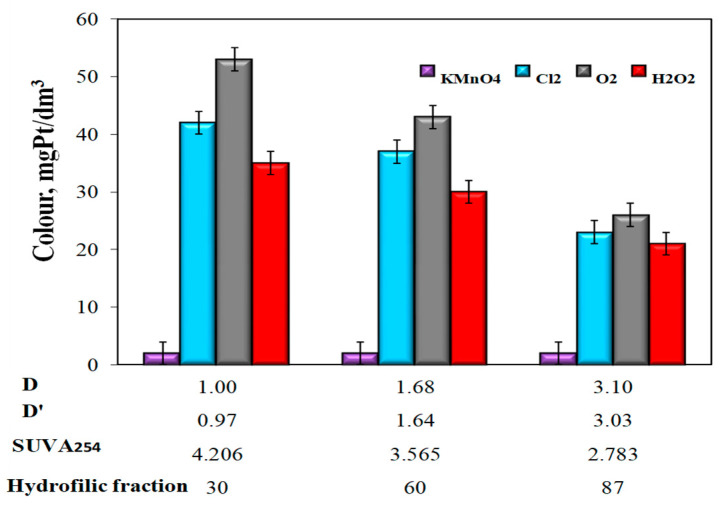
The impact of the type of oxidant and values of the co-occurrence coefficient of organic substances and total iron (D and D’), SUVA_254_ coefficient, and the content of hydrophilic organic substances (%) on the apparent color of water.

In water samples characterized by the highest value of the co-occurrence coefficient of organic substances and total iron (D = 3.0 and D’ = 3.03), the lowest value of SUVA_254_ (2.783 m^2^/gC, and the highest content of 87% hydrophilic organic substances, the highest Fe(II) oxidation efficiency to Fe (III) (η = 96%, at pH = 7.18) was obtained using potassium permanganate and the smallest after oxidation with hydrogen peroxide (η = 43%, at pH = 7.11) (Figure 3). The use of KMnO_4_ for the oxidation of Fe(II) caused a significant decrease in the apparent color of water, while oxidation with oxygen and other chemical oxidants (hydrogen peroxide and chlorine water) caused a clear increase in the apparent color of water in relation to raw water, which for Raw Waters 1, 2 and 3 were respectively 10, 15, and 19 mgPt/dm^3^ (Figure 4, Table 3). 

**Figure 5:**
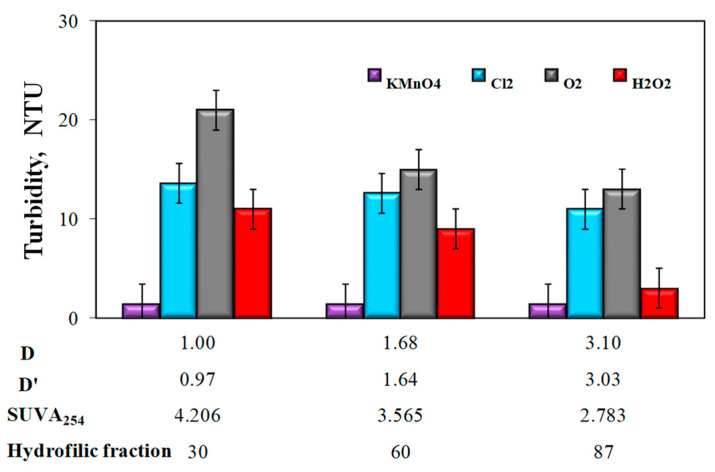
The influence of the type of oxidant and values of the co-occurrence coefficient of organic substances and total iron (D and D’), SUVA_254_ coefficient, and the content of hydrophilic organic substances (%) on water turbidity.

The analysis of the obtained test results also showed that the apparent color intensity (Figure 4) and the turbidity of water (Figure 5) after the aeration or oxidation with chemical oxidants (except for potassium permanganate) decreased with the increasing value of the coexistence of organic substances and total iron D and D‘, along with a decrease in the SUVA_254_ value and an increase in the raw water content of hydrophilic organic substances. 

The presented test results indicated the inhibition of Fe(II) to Fe(III) oxidation by organic compounds present in water when using aeration, hydrogen peroxide, and chlorine water. This regularity was not found using KMnO_4_ as an oxidant. At the working pH = 7, oxygen has an oxidation potential of 0.82 V, while chlorine water 1.28 V, permanganate 0.86 V, and hydrogen peroxide 1.37 V. The lowest efficiency of Fe(II) oxidation with hydrogen peroxide, even though it had the highest oxidation potential of 1.37 V, was due to the fact that hydrogen peroxide in water is very slow to act and is also a relatively weak oxidant with respect to organic compounds. This is due to the relatively low reactivity of H_2_O_2_ towards organic functional groups and the fact that in the presence of electrophilic compounds, it can react as a nucleophile (undergo substitution reactions), without manifesting oxidizing properties [33]. Although the kinetics of hydrogen peroxide reactions with water pollutants, especially organic pollutants, is relatively low, hydrogen peroxide presents a high capacity to be combined with other oxidants and agents (catalysts or radiation) to initiate radical chain mechanisms that lead to the formation of hydroxyl radicals [58]. The oxidation mechanisms for hydrogen peroxide and potassium permanganate are quite different. Potassium permanganate often provides more rapid destruction of specific compounds than hydrogen peroxide. According to many authors [25,26,43], KMnO_4_ reacts fast with iron(II) also in the presence of organic substances, easily breaking carbon-carbon double bonds and oxidizing functional groups that are responsible for the color and taste of water. At the working pH = 7, potassium permanganate has a lower oxidizing potential (0.86 V) than hydrogen peroxide (1.37 V) and chlorine water (1.28 V). In an environment with a neutral reaction, KMnO_4_ is reduced to MnO_2_. According to the literature [45], the precipitating MnO_2_ during oxidation with KMnO_4_ catalyzes the oxidation process of Fe (II) and Mn(II) according to Reactions 4 and 5. 

2Fe^2+^ + 2MnO_2_ + 5H_2_O ↔ 2Fe(OH)_3_ + Mn_2_O_3_ + 4H_2_O (4)

Mn^2+^ + MnO_2_ + H_2_O_2_ ↔ Mn_2_O_3_ + 2H^+^ (5)

Some researchers have expressed the opinion that the utilization of KMnO_4_ to break the organic molecules can also make the organic substance much less tenacious as a complexing agent [25,26].

### 2.3. Oxidation and Sedimentation Efficiency

**Figure 6:**
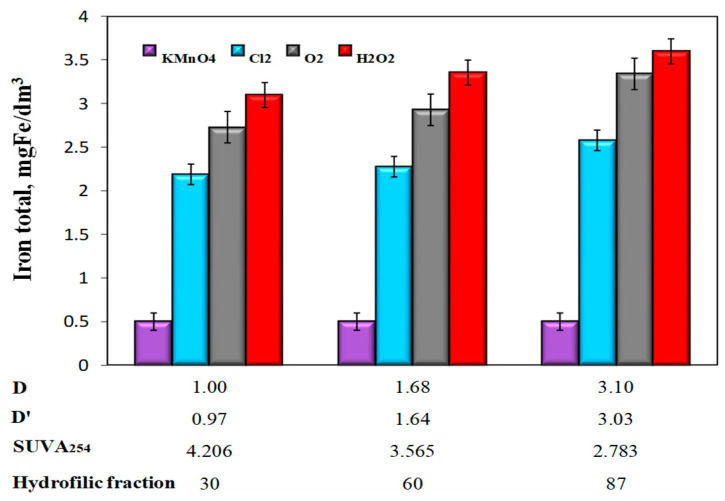
The influence of the type of oxidant and values of the co-occurrence coefficient of organic substances and total iron (D and D‘), SUVA_254_ coefficient value, and the content of hydrophilic organic substances (%) on the concentration of total iron in water after sedimentation.

Analyzing the obtained test results showed that even in water samples with the lowest value of the co-occurrence coefficient of organic substances and total iron D = 1 and D’ = 0.97, despite the high of 49% (for H_2_O_2_), 1 h sedimentation did not ensure the removal of iron compounds required for water intended for human consumption (≤0.2 mgFe/dm^3^). The lowest concentrations of total iron in water samples after oxidation and sedimentation were obtained after the use of potassium permanganate and the highest after oxidation with hydrogen peroxide (Figure 6). 

The effect of the co-occurrence coefficients of organic substances and total iron D and D’ as well as the specific absorbance value SUVA_254_ on the degree of total iron removal was only unambiguous in the case of aeration and oxidation with hydrogen peroxide and chlorine water. Together with the increase in the values of the co-occurrence coefficients of organic substances and total iron D and D’, as well as the decrease in the SUVA_254_ value, and thus the increase in the raw water content of hydrophilic organic substances, the efficiency of removing iron compounds from water decreased for these oxidants (Table 4).

In samples of water after oxidation with KMnO_4_ the highest iron removal efficiency was found to be 86%. When using this oxidizer, no impact was found of the values of D and D’ coefficients, as well as the type of organic matter fraction (hydrophilic, hydrophobic) upon the degree of total iron removal (Table 4). All water samples after oxidation and sedimentation, with the exception of samples after oxidation with KMnO_4_, were characterized by a greater intensity of color than raw water. Only in water samples with the highest D and D’ coefficients after aeration and hydrogen peroxide oxidation, the color exceeded 15 mgPt/dm^3^ and was 24 mgPt/dm^3^ (after aeration) and 28 mgPt/dm^3^ (after H_2_O_2_ oxidation). Regardless of the type of oxidizer used in all water samples, after oxidation and sedimentation the turbidity exceeded 1NTU, i.e., the limit value for water intended for human consumption. The lowest turbidity of 2 NTU was found in all samples of water after oxidation with KMnO_4_ and the highest 22 NTU in water after H_2_O_2_ oxidation characterized by the highest value of the co-occurrence coefficient of organic substances and total iron D = 3.10 and D’ = 3.03 and the lowest value SUVA_254_ (Table 5).

**Figure 7:**
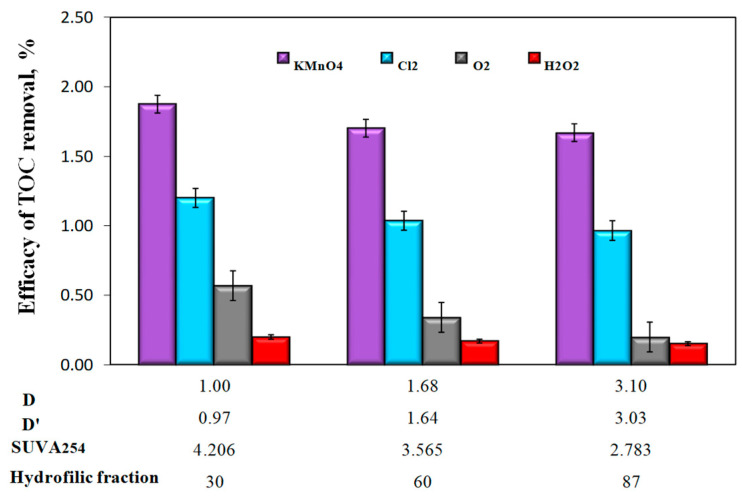
The influence of the type of oxidant and values of the co-occurrence coefficient of organic substances and total iron (D and D’), SUVA_254_ coefficient value, and hydrophilic organic content (%) on the efficiency of TOC removal from water in the sedimentation process.

Analysis of the test results also showed that changes in the content of organic substances in the water samples after oxidation and sedimentation were minimal (Figure 7), which indicates that mainly inorganic iron compounds were sedimented, and finely dispersed iron compounds, insusceptible to sedimentation, stabilized by organic substances, remained in the water. The highest TOC removal efficiency of only 1.90% was found after oxidation with potassium permanganate in water with the lowest value of the co-occurrence coefficients of organic substances and iron compounds (D = 1 and D’ = 0.97) and characterized by the lowest content of hydrophilic organic substances. The lowest TOC removal efficiency was found in all water samples after oxidation with hydrogen peroxide (Figure 7).

**Figure 8:**
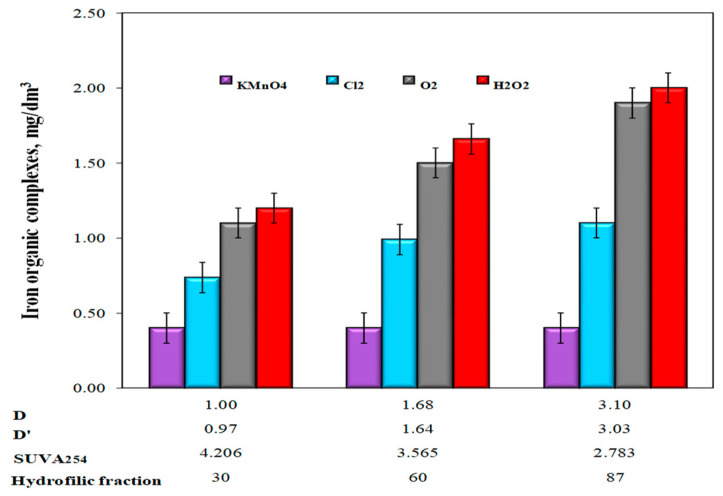
The influence of the type of oxidant and values of the co-occurrence coefficient of organic substances and total iron (D and D’), SUVA_254_ value, and the content of hydrophilic organic substances (%) on the amount of iron-organic complexes in water after the sedimentation process.

The highest effectiveness of water purification after the use of KMnO_4_ was due to the adsorption and catalytic properties of precipitating manganese oxide (IV), which also improved the sedimentation properties of the resulting oxidation products. Several researchers [2,16,23,38,42,43,45,48] suggested that the use of KMnO_4_ should be right for oxidizing Fe(II) occurring in compounds with organic substances because the oxidation process is additionally aided by the adsorptive and catalytic properties of the precipitated manganese oxide (IV). The MnO_2_ that appears has a vast specific surface area with good sorptive characteristics [42,43,44,45]. Some researchers have expressed the opinion that utilization of KMnO_4_ to break the organic molecules, thus reducing the color, can also make the organic substance much less tenacious as a complexing agent [23]. According to Teh Fu Yen [28], the catalytic oxidation of Fe(II) ions with KMnO_4_ allows its dose to be reduced below the stoichiometric amount, but only if there are no other reduced substances in the water. During the studies, the manganese concentration in water after oxidation and sedimentation processes was also monitored, mainly due to the dosage of potassium permanganate. Removal of manganese compounds from water at a level of approximately 15% was found only in water after oxidation with KMnO_4_. In the remaining water samples after aeration and oxidation with other tested chemical oxidants, regardless of the value of the D and D’ coefficients in raw water, no removal of manganese from water was found. According to the literature [45], the precipitating MnO_2_ during oxidation with KMnO_4_ catalyzes the oxidation process of Mn(II) with oxygen dissolved in water and its adsorption. The analysis of the dependencies presented in Figure 8 showed that after oxidation with hydrogen peroxide, dissolved oxygen, and chlorine oxidant, the number of organo-ferrous connections in purified water increased with the increase of the co-occurrence coefficients of organic substances and total iron D and D’, as well as lowering SUVA_254_ and increasing the content of hydrophilic organic substances in raw water. The lowest concentrations of iron bound to organic substances were found in water after the oxidation process with KMnO_4_ and the highest after the oxidation process with hydrogen peroxide (Figure 8). Only when KMnO_4_ was used as the oxidant, the number of organic iron compounds in purified water did not depend on the values of the co-occurrence coefficients of organic substances and iron compounds (D and D’) in raw water, SUVA_254_ coefficient, and the content of the raw fraction of organic substances of the hydrophilic type (Figure 8). 

In water samples after oxidation with hydrogen peroxide, a slight increase in the concentration of dissolved organic carbon in relation to raw water was found. For Waters 1, 2, and 3, the concentration of dissolved organic carbon increased by 0.150, 0.250, and 0.400 mgC/dm^3^, respectively. According to Teh Fu Yen [28], the mechanism of oxidation with hydrogen peroxide consists of fractionation of large organic molecules into smaller ones. This destroys the conjugated bonds that determine the color of organic substances such as humic substances, but this is not always synonymous with the breakdown of organo-iron complexes and the release of iron ions into the solution, and above all, the removal of organic ligands from water. The intermediate organic oxidation products of organic compounds remaining in the water can still stabilize iron by forming organo-ferrous connections, for which sedimentation and accelerated filtration are not enough. The dependency analysis presented in Figure 8 also showed that large amounts of organo-ferrous compounds were found in water samples after the aeration process, in which the largest increase in pH to 8.40 (pH = 7.18—KMnO_4_; pH = 7.25—chlorine oxidant; pH = 7.20—H_2_O_2_) was also found. At pH ≥8, in the case of organic substances, such as, for example, humic substances, these substances are completely dissociated. At pH = 4.6–4.9, dissociation of protons from carboxyl groups starts, and at pH ≥8, protons dissociate from hydroxyl groups [23,24,25,26]. An increase in the degree of dissociation of organic substances leads to a decrease in the efficiency of the removal of metals from water as a result of the formation of metal-organic compounds. In the case of groundwater, colored iron-organic complexes can form, especially with humic substances. Based on the literature information on the kinetics of Fe(II) oxidation with potassium permanganate and chlorine oxidants, it can be concluded that these reactions occur quickly at pH ≥ 7 [22,23,25]. In waters that are hardly susceptible to iron removal, organic substances’ oxidation of Fe(II) ions using chlorine oxidants, and mainly chlorine, does not always lead to the formation of well-sedimenting iron(III) hydroxide agglomerates susceptible to retention in the filter bed. According to Ghernaout et al. [12], when using hydrogen peroxide, the optimal pH value for the oxidation of most organic substances in the presence of Fe(II) ions is 4, and the oxidation time and dose of hydrogen peroxide depend on the type of oxidized compound and the concentration of Fe(II) ions. Too high concentrations of Fe(II) ions in water may reduce the effectiveness of oxidation with hydrogen peroxide acting as scavengers of hydroxyl radicals ^•^OH. In the conducted tests, the lowest efficiency of removing iron compounds from groundwater after oxidation with hydrogen peroxide (Figure 6 and Figure 8) could have been the result of using too low a dose of hydrogen peroxide and too high a pH value of water, which determines the form of the presence of the oxidant and different reaction mechanisms in acid and alkaline environments. According to literature reports [40], the use of appropriate oxidation parameters with Fenton’s reagent (pH = 4; Fe(II): H_2_O_2_ = 1:5) ensures 90% removal efficiency from DOC with molecular weight > 0.5 kDa. However, this method is not commonly used for purifying water intended for human consumption due to the need to acidify the water and remove the Fe(III) ions formed. 

## 3. Materials and Methods 

### 3.1. Groundwater Samples

The study was conducted with the use of three types of groundwater from Quaternary deposits significantly differing in the content of organic substances. The groundwater intake is located in Poland in the area of the Warsaw-Berlin Urstromtal, 3–4 km from the shoreline of the river, in the area of the Odra river oxbow. They are 22 wells, 19 to 30 m deep, located near peat bogs supplying the Zawada water treatment plant together with the water from the Obrzyca river. The groundwater was also characterized by an increased concentration of total iron and manganese. Part of the iron found in the groundwater formed organometallic connections with organic substances. The groundwater sample characteristics are given in Table 3.

### 3.2. Experimental Procedure and Analytical Methods 

The scope of study included determining the effectiveness of water purification, with particular emphasis on removing iron compounds from the three groundwaters significantly differing in content and type of organic substances in the following technological systems: aeration, flocculation, and sedimentation, chemical oxidation (KMnO_4_, H_2_O_2_, Cl_2_), flocculation, and sedimentation.

In order to oxidize Fe(II), the water was aerated for 15 min, or chemical oxidants were dosed: potassium permanganate, chlorine water, and hydrogen peroxide. In the studies, due to the oxidation of Fe(II), a stoichiometric dose of chemical oxidants was used, and the oxidation time was 5 min. After aeration or chemical oxidation, twenty minutes of flocculation (at a rotational speed of 30 rpm) and 1 h sedimentation were used. The oxidation-reduction potentials for the tested oxidants for the working pH = 7 were calculated. The calculated oxidation-reduction potentials were 1.37, 1.28, 0.86, and 0.82 V, respectively, for hydrogen peroxide, chlorine water, potassium permanganate, and oxygen. Consider that chlorine added to water undergoes a disproportionate reaction [33]:

Cl_2_ + H_2_O ↔ H^+^ + HOCl + Cl^−^ (6)

The criterion of effective water purification was the reduction of the values of the tested water quality indicators to the limit values in water intended for human consumption, specified in the Regulation of the Minister of Health [59]. The fractionation method using XAD7HP and XAD4 polymer adsorbents was used to characterize the organic substances found in the tested groundwater. Using this technique, the dissolved organic compounds were separated into highly hydrophobic (isolated on XAD7HP resin), weakly hydrophobic (isolated on XAD4 resin), and hydrophilic (not adsorbed on any resin) fractions [53]. Infrared (IR) spectroscopy and the specific absorbance index SUVA_254_ were also used to assess the quality of organic substances found in the investigated groundwater. Specific absorbance values in UV-SUVA_254_ were calculated using the formula SUVA_254_ = $\frac{\mathrm{UV}254\mathrm{nm}}{\mathrm{DOC}}\;$(m^2^/gC) where UV_254_ is absorbance at 254 nm (m^−1^) and DOC dissolved organic carbon (gC/m^3^) [10,13]. To assess the degree of organic contamination of water samples and its impact on the course and efficiency of the processes studied, the co-occurrence coefficient of organic substances and total iron was also used, calculated as D = TOC/Fe_tot_ (mgC/mgFe) and D’ = DOC/Fe_tot_ (mgC/mgFe). The organic substances in all samples were determined by measuring absorbance at 254 nm and total (TOC) and dissolved organic carbon (DOC) concentration. The TOC and DOC were measured using the thermal method and a Shimadzu TOC analyzer (Hadano, Kanagawa, Japan). UV absorbance at 254 nm was measured by a UV-Vis spectrophotometer Agilent Cary 60 (Santa Clara, California, USA). Organic substances contained in groundwater were fractionated. The groundwater was filtered through a 0.45 µm filter for DOC determination. The fraction containing substances <0.45 μm was separated on polymeric adsorbents Amberlite XAD7HP and XAD4 from Rohm & Haas (Miami Beach, FL, USA) in order to isolate the hydrophobic, hydrophilic, and intermediate transfill fraction according to the fractionation procedure given by Aiken et al. [53]. Humic substances were also extracted from groundwater samples after filtration through a 0.45 μm filter according to the methodology developed by Aiken et al. [54] using different solubility in acids and alkalis of individual fractions of humic substances. Humic substances extracted from raw water were analyzed using infrared (IR) spectroscopy using a FTIR THERMO SCIENTIFIC NICOLET iS50 spectrometer (Waltham, MA, USA) operating in the NIR-, MID-, and FAR-IR ranges using the KBr compensation tablet technique. Color and apparent color were indicated in accordance with ISO 7887-Method C [60], using a spectrophotometer Agilent Cary 60 (Santa Clara, CA, USA). Color was determined after filtration of the water sample through a membrane filter of pore size 0.45 μm. Apparent color was determined without filtration of the water sample through a membrane filter. Color and apparent color of the sample were calculated using the following equation: $C=\frac{A410}{a\cdot d}$ (mg Pt/dm^3^) where C is the true or apparent color of the sample, A_410_ the absorbance of the sample at λ = 410 nm, a the specific absorption coefficient of the calibration solution of potassium hexachloroplatine and cobalt chloride (mm^−1^(mg Pt/dm^3^)^−1^), and d the optical pathlength (mm) [61]. The total iron and iron(II) concentrations were determined with the Agilent Cary 60 spectrophotometer (Santa Clara, California, USA) using the 1,10 phenanthroline method. Iron fixed with organic substances was determined as the difference between the iron concentration determined in the post-mineralization test and without mineralization, taking into account the amount of iron-inorganic bonds. Manganese concentrations were determined with atomic emission spectroscopy (ISP-OES, 5300DV, Perkin Elmer Company, U.S.). The dissolved oxygen and pH of the groundwater were determined with an WTW Multi Line P4. Turbidity was measured using the Hach 2100N Turbidimeter (Brønshøj, Germany). The alkalinity was determined with a titrimetric method against methyl orange using 0.1 M aqueous solutions of HCl. 

## 4. Conclusions

The research presented in this paper allowed us to draw the following conclusions:

With the rise of the co-occurrence coefficients of organic substances and total iron (D = TOC/Fe_tot_ and D’ = DOC/Fe_tot_), as well as with the increase in the content of dissolved organic hydrophilic substances containing aromatic rings, among which fulvic and humic acids were present, the amount of colored organo-ferrous connections in raw water increased and so did the percentage of Fe(II) in total iron. The regularities found testify to the iron stabilization by organic substances and the inhibition of Fe(II) oxidation. 

The type of oxidizer used, as well as the type and content of organic substances present in raw water determined the effectiveness of Fe(II) oxidation and the removal of iron in the sedimentation process. The usefulness of the tested oxidants decreased in accordance with the following series: potassium permanganate (oxidation power 0.86 V at pH = 7) > chlorine water (oxidation power 1.28 V at pH = 7) > dissolved oxygen (oxidation power 0.82 V at pH = 7) > hydrogen peroxide (oxidation power 1.37 V at pH = 7). The lowest efficiency of Fe(II) oxidation with hydrogen peroxide even though it had the highest oxidation potential of 1.37 V was due to the fact that hydrogen peroxide in water is very slow to act and is also a relatively weak oxidant with respect to organic compounds. This is due to the relatively low reactivity of H_2_O_2_ towards organic functional groups and the fact that in the presence of electrophilic compounds, it can react as a nucleophile, without manifesting oxidizing properties.

Together with the increasing value of the co-occurrence coefficients of organic substances and total iron (D = TOC/Fe_tot_ and D’ = DOC/Fe_tot_) and with the increase in the content of dissolved hydrophilic organic substances containing aromatic rings in the raw water, the efficiency of Fe(II) oxidization and removal of iron in the sedimentation process decreased. Such a regularity was not found using only KMnO_4_ as an oxidant. 

The lowest concentrations of organo-ferrous connections were found in the water after oxidation with KMnO_4_, and the highest number of organo-ferrous connections and an increase in the concentration of dissolved organic carbon were found in the water after oxidation with H_2_O_2_. High concentrations of organo-ferrous connections were also found in aerated water samples. Therefore, it was shown that the formation of organo-ferrous connections is conducive to the fractionation of large particles of organic substances into smaller ones as a result of the use of chemical oxidants such as H_2_O_2_ or increasing the water pH to ≥ 8 as a result of aeration, which could cause complete dissociation of organic substances and thus create good conditions for the formation of colorful hard-sedimenting organo-ferrous connections. 

The best effects of Fe(II) oxidation and removal of iron from water containing organic substances were obtained using KMnO_4_, although it has a lower oxidation-reduction potential than hydrogen peroxide and chlorine water (at pH = 7), due to MnO_2_ precipitating from water catalyzing the oxidation of Fe(II) and acting as a load and adsorbent of the resulting oxidation products. Only when KMnO_4_ was used as the oxidant, the number of organic iron connections remaining in the purified water after the sedimentation process did not depend on the co-occurrence coefficients of organic substances and iron compounds (D = TOC/Fe_tot_ and D’ = DOC/Fe_tot_) and the content in water of the raw fraction of hydrophilic organic substances. Therefore, in the case of groundwater purification in which there are organo-ferrous connections, it is advisable to use KMnO_4_, which, according to literature reports, also does not cause the formation of oxidation by-products having negative effects on the human body. 

## Figures and Tables

**Table 1:** Share of the Natural Organic Matter (NOM) fractions on groundwaters.

Fraction	Water 1	Water 2	Water 3
	Percentage, %		
Hydrophobic fraction	60	35	10
Hydrophilic fraction	30	60	87
Intermediate transfill fraction	10	5	3

**Table 2:** Correlations between the indicators of the quality of groundwater samples.

Equation of Linear Correlation	Pearson Correlation Coefficient (r)
Fe org. complexes = 1.2019 D − 0.848	0.9988
Fe org. complexes = 1.2256 D’ − 0.8364	0.9988
Fe org. complexes = 0.167 UV_254_ − 2.1118	0.9900
Fe org. complexes = 7.7423 − 1.7846 SUVA_254_	0.9824
Color= 2.2326 D + 2.3652	0.9506
Color = 2.2779 D’ + 2.3843	0.9511
Color = 0.3213 UV_254_ − 0.2193	0.9757
Color = 18.943 − 3.4917 SUVA_254_	0.9848
Turbidity = 3.2562 Fe(III) + 4.3993	0.9564
Turbidity = 12.526 − 2.8941D	0.9390
Turbidity = 12.502 − 2.9529 D’	0.9397
Turbidity = 15.908 − 0.418 UV_254_	0.9674
Fe(II)/Fe_tot_ = 20.715 D + 37.756	0.8060
Fe(II)/Fe_tot_ = 21.148 D’ + 37.908	0.8070
Fe(II)/Fe_tot_ = 21.148 UV_254_ + 11.375	0.9500

**Table 3:** Value ranges analyzed for the groundwater quality parameters.

Indicator	Unit	Value		
		Water 1	Water 2	Water 3
pH	-	7.27 ± 0.2	7.25 ± 0.2	7.18 ± 0.2
Dissolved Oxygen	mgO_2_/dm^3^	0.55 ± 0.02	0.55 ± 0.02	0.50 ± 0.02
Alkalinity	mmol/dm^3^	3.60 ± 0.05	3.50 ± 0.05	3.40 ± 0.05
Turbidity	NTU	10.50 ± 0.1	6.40 ± 0.1	4.00 ± 0.1
Color	mgPt/dm^3^	4 ± 1	7 ± 1	9 ± 1
Apparent Color	mgPt/dm^3^	10 ± 1	15 ± 1	19 ± 1
TOC	mgC/dm^3^	3.500 ± 0.05	5.900 ± 0.05	10.80 ± 0.05
DOC	mgC/dm^3^	3.400 ± 0.05	5.750 ± 0.05	10.60 ± 0.05
UV_254_	m^−1^	14.300 ± 0.200	20.500 ± 0.200	29.500 ± 0.200
Iron total	mgFe/dm^3^	3.500 ± 0.100	3.500 ± 0.100	3.500 ± 0.100
Iron (II)	mgFe/dm^3^	1.600 ± 0.100	3.200 ± 0.100	3.350 ± 0.100
Iron (III)	mgFe/dm^3^	1.900 ± 0.100	0.300 ± 0.010	0.150 ± 0.010
Fe(II)/Fe_tot_	%	46	91	96
Iron fixed in metal organic complexes	mg/dm^3^	0.400	1.100	2.900
Manganese	mgMn/dm^3^	0.200 ± 0.020	0.200 ± 0.020	0.200 ± 0.020
D = TOC/Fe_tot_	mgC/mgFe	1.00	1.68	3.10
D’ = DOC/Fe_tot_	mgC/mgFe	0.97	1.64	3.03
SUVA_254 =_ $\frac{\mathrm{UV}254\mathrm{nm}}{\mathrm{DOC}}$	m^2^/gC	4.206	3.565	2.783

**Table 4:** The impact of the type of oxidant, values of the D (TOC/Fe_tot_) and D’ (DOC/Fe_tot_) coefficients as well as the values of SUVA_254_ and hydrophilic organic content (%) coefficient on the efficiency of removal of total iron from groundwater in oxidation and sedimentation processes.

D	D’	SUVA_254,_ m^2^/gC	Hydrophilic Fraction of Organic Substances, %	The Efficiency of Removal of Total Iron, %			
				Type of Oxidant			
				O_2_	KMnO_4_	Chlorine Water	H_2_O_2_
1.00	0.97	4.206	30	23	86	38	12
1.68	1.64	3.565	60	18	86	30	6
3.10	3.03	2.783	87	4	86	26	3

**Table 5:** The influence of the type of oxidant, values of the coefficients D (TOC/Fe_tot_) and D’ (DOC/Fe_tot_) and the SUVA_254_ on the real color and turbidity in water after oxidation and sedimentation processes.

D and D’	SUVA_254_	Hydrophilic Fraction of Organic Substances, %	Type of Oxidant									
			Raw Water	O_2_	KMnO_4_	Chlorine Water	H_2_O_2_	Raw Water	O_2_	KMnO_4_	Chlorine Water	H_2_O_2_
			Color, mgPt/dm^3^					Turbidity, NTU				
1.00 0.97	4.206	20	4	6	2	6	11	10.50	12	2	3	14
1.68 1.64	3.565	60	7	9	3	9	14	6.40	13	2	10	15
3.10 3.03	2.783	87	9	24	5	11	28	4.0	14	2	12	22
